# Tetra­chlorido(4,4′-dimethyl-2,2′-bipyridine-κ^2^ *N*,*N*′)platinum(IV)

**DOI:** 10.1107/S1600536808016796

**Published:** 2008-06-13

**Authors:** Leila Hojjat Kashani, Vahid Amani, Mohammad Yousefi, Hamid Reza Khavasi

**Affiliations:** a Islamic Azad University, Shahr-e-Rey Branch, Tehran, Iran; b Department of Chemistry, Shahid Beheshti University, Tehran 1983963113, Iran

## Abstract

The asymmetric unit of the title compound, [PtCl_4_(C_12_H_12_N_2_)], contains one half-mol­ecule; a twofold rotation axis passes through the Pt atom and the mid-point of the C—C bond linking the two rings. The Pt^IV^ atom is six-coordinated in an octa­hedral configuration by two N atoms of the 4,4′-dimethyl-2,2′-bipyridine ligand and four terminal Cl atoms. In the crystal structure, there are weak π–π inter­actions between pyridine rings, with a centroid–centroid distance of 4.365 (3) Å.

## Related literature

For related literature, see: Hedin (1886 ▶); Joergensen (1900 ▶); Bajusz *et al.* (1989 ▶); Vorobevdesyatovskii *et al.* (1991 ▶); Gaballa *et al.* (2003 ▶); Casas *et al.* (2005 ▶); Hambley (1986 ▶); Hafizovic *et al.* (2006 ▶); Delir Kheirollahi Nezhad *et al.* (2008 ▶); Crowder *et al.* (2004 ▶); Junicke *et al.* (1997 ▶); Khripun *et al.* (2006 ▶); Witkowski *et al.* (1997 ▶); Kuduk-Jaworska *et al.* (1988 ▶, 1990 ▶); Bokach *et al.* (2003 ▶); Kukushkin *et al.* (1998 ▶); Garnovskii *et al.* (2001 ▶); Luzyanin, Kukushkin *et al.* (2002 ▶); Gonzalez *et al.* (2002 ▶); Luzyanin, Haukka *et al.* (2002 ▶); Yousefi *et al.* (2007 ▶).
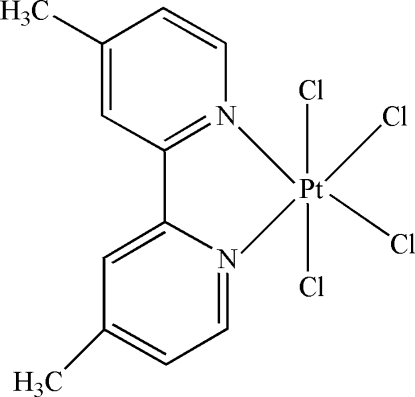


## Experimental

### 

#### Crystal data

[PtCl_4_(C_12_H_12_N_2_)]
*M* *_r_* = 521.12
Orthorhombic, 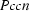

*a* = 6.9497 (7) Å
*b* = 13.3774 (13) Å
*c* = 17.3195 (16) Å
*V* = 1610.2 (3) Å^3^
*Z* = 4
Mo *K*α radiation
μ = 9.36 mm^−1^
*T* = 298 (2) K
0.25 × 0.23 × 0.21 mm

#### Data collection

Stoe IPDS II diffractometer
Absorption correction: numerical [shape of crystal determined optically (*X-SHAPE* and *X-RED*; Stoe & Cie, 2005 ▶)*T*_min_ = 0.172, *T*_max_ = 0.275
5803 measured reflections
2157 independent reflections
1779 reflections with *I* > 2σ(*I*)
*R*_int_ = 0.049

#### Refinement

*R*[*F* ^2^ > 2σ(*F* ^2^)] = 0.038
*wR*(*F* ^2^) = 0.097
*S* = 1.16
2157 reflections
87 parameters
H-atom parameters constrained
Δρ_max_ = 0.95 e Å^−3^
Δρ_min_ = −0.81 e Å^−3^

### 

Data collection: *X-AREA* (Stoe & Cie, 2005 ▶); cell refinement: *X-AREA*; data reduction: *X-RED* (Stoe & Cie, 2005 ▶); program(s) used to solve structure: *SHELXS97* (Sheldrick, 2008 ▶); program(s) used to refine structure: *SHELXL97* (Sheldrick, 2008 ▶); molecular graphics: *ORTEP-3 for Windows* (Farrugia, 1997 ▶); software used to prepare material for publication: *WinGX* (Farrugia, 1999 ▶).

## Supplementary Material

Crystal structure: contains datablocks I, global. DOI: 10.1107/S1600536808016796/hk2470sup1.cif

Structure factors: contains datablocks I. DOI: 10.1107/S1600536808016796/hk2470Isup2.hkl

Additional supplementary materials:  crystallographic information; 3D view; checkCIF report

## Figures and Tables

**Table:** 

Pt1—N1	2.031 (4)
Pt1—Cl2	2.3038 (13)
Pt1—Cl1	2.3146 (16)

**Table:** 

N1—Pt1—N1^i^	80.4 (2)
N1—Pt1—Cl2	175.58 (12)
N1—Pt1—Cl2^i^	95.40 (13)
Cl2—Pt1—Cl2^i^	88.85 (8)
N1—Pt1—Cl1^i^	87.72 (14)
Cl2—Pt1—Cl1^i^	90.96 (6)
N1—Pt1—Cl1	89.81 (14)
Cl2—Pt1—Cl1	91.34 (6)
Cl1^i^—Pt1—Cl1	176.78 (8)

Symmetry code: (i) 
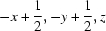
.

**Fig. 1.**
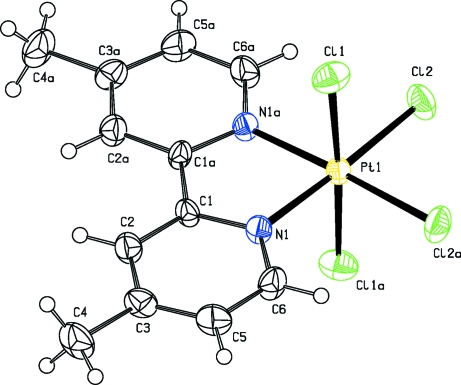
The molecular structure of the title molecule, with the atom-numbering scheme. Displacement ellipsoids are drawn at the 40% probability level [symmetry code: (a) 1/2 - x, 1/2 - y, z].

## supplementary crystallographic information

### Comment

Amine platinum(IV) complexes have been known since the end of the last century (Hedin, 1886; Joergensen, 1900). Some of them have cancerostatic properties from which new interest aroused in these complexes (Bajusz *et al.*, 1989; Vorobevdesyatovskii *et al.*, 1991). Due to the kinetic inertness of hexachloro -platinate(IV), *cis*- and *trans*-[PtC1_4_*L*_2_] complexes (*L*=N, O, P, S donor ligand) were mainly prepared by oxidation reactions of the corresponding platinum(II) complexes [PtCl_2_*L*_2_] (Hedin, 1886; Joergensen, 1900).

Several Pt^IV^ complexes, with formula, [PtCl_4_(N-N)], such as [PtCl_4_- (bipyi)], (II), (Gaballa *et al.*, 2003), [PtCl_4_(Me_2_bim)], (III), (Casas *et al.*, 2005), [PtCl_4_(bipy)], (IV), (Hambley, 1986), [PtCl_4_(dcbipy)].H_2_O, (V), (Hafizovic *et al.*, 2006), [PtCl_4_{pz(py)_2_}], (VI), (Delir Kheirollahi Nezhad *et al.*, 2008) and [PtCl_4_(dpk)], (VII), (Crowder *et al.*, 2004) [where bipyi is 2,2'-bi-pyrimidinyl, Me_2_bim is 1,1'-dimethyl- 2,2'-bi-imidazolyl, bipy is 2,2'-bipyridine, dcbipy is 2,2'- bipyridine-5,5'-dicarboxylic acid, pz(py)_2_ is 2,3-bis(2-pyridyl)pyrazine and dpk is bis(2-pyridyl)ketone] have been synthesized and characterized by single-crystal X-ray diffraction methods.

There are also several Pt^IV^ complexes, with formula, [PtCl_4_*L*_2_], such as *cis*- and *trans*-[PtCl_4_(py)_2_], (VIII), (Junicke *et al.*, 1997), *cis*- and *trans*-[PtCl_4_(PzH)_2_], (IX), (Khripun *et al.*, 2006), *trans*-[PtCl_4_(NH_3_)_2_](1-Mu), (X), (Witkowski *et al.*, 1997), *trans*-[PtCl_4_(1-Prim)_2_], (XI), (Kuduk-Jaworska *et al.*, 1988), *cis*-[PtCl_4_(1-Etim)_2_], (XII), (Kuduk-Jaworska *et al.*, 1990), *trans*-[PtCl_4_{NH=C(NMe_2_)OH}_2_], (XIII), (Bokach *et al.*, 2003), *trans*-[PtCl_4_{NH=C(Me)ON=CMe_2_}_2_], (XIV), (Kukushkin *et al.*, 1998), *cis*-[PtCl_4_{NH=C(Et)N=CPh_2_}_2_], (XV), (Garnovskii *et al.*, 2001), *trans*- [PtCl_4_{NH=C(Et)ON=C(OH)Ph}_2_].2DMSO, (XVI), (Luzyanin, Kukushkin *et al.*, 2002), *trans*-[PtCl_4_{NH=C(OMe)Bu*^t^*}_2_], (XVII), (Gonzalez *et al.*, 2002), *trans*-[PtCl_4_{NH=C(OH)Et}_2_], (XVIII), (Luzyanin, Haukka *et al.*, 2002) and *trans*- [PtCl_4_(pz)_2_], (XIX), (Yousefi *et al.*, 2007) [where PzH is pyrazole, 1-Mu is 1-methyluracil, 1-Prim is 1-propylimidazole, 1-Etim is 1-ethylimidazoyl and Pz is pyrazine] have been synthesized and characterized by single-crystal X-ray diffraction methods. We report herein the synthesis and crystal structure of the title compound, (I).

The asymmetric unit of (I) (Fig. 1) contains one-half molecule. The Pt^IV^ atom is six-coordinated in octahedral configuration (Table 1) by two N atoms of 4,4'-dimethyl-2,2'-bipyridine ligand and four terminal Cl atoms. The Pt-Cl and Pt-N bond lengths and angles (Table 1) are in good agreement with the corresponding values in (II), (III), (V) and (VI).

In the crystal structure, weak π—π interactions between pyridine rings [symmetry code: 3/2 - x, 1/2 - y, z] may be effective in the stabilization of the structure, with a centroid-centroid distance of 4.365 (3) Å.

### Experimental

For the preparation of the title compound, a solution of 4,4'-dimethyl-2,2' -bipyridine (0.11 g, 0.58 mmol) in methanol (10 ml) was added to a solution of H_2_PtCl_6_.6H_2_O, (0.30 g, 0.58 mmol) in methanol (10 ml) at room temperature. Crystals suitable for X-ray analysis were obtained by methanol diffusion in a solution of yellow precipitate in DMSO after one week (yield; 0.25 g, 82.8%).

### Refinement

H atoms were positioned geometrically, with C-H = 0.93 and 0.96 Å for aromatic and methyl H and constrained to ride on their parent atoms with U_iso_(H) = 1.2U_eq_(C).

### Crystal data

**Table:**

[PtCl_4_(C_12_H_12_N_2_)]	*F*_000_ = 976
*M**_r_* = 521.12	*D*_x_ = 2.150 Mg m^−^^3^
Orthorhombic, *P**c**c**n*	Mo *K*α radiation λ = 0.71073 Å
Hall symbol: -P 2ab 2ac	Cell parameters from 1510 reflections
*a* = 6.9497 (7) Å	θ = 2.8–29.2º
*b* = 13.3774 (13) Å	µ = 9.36 mm^−^^1^
*c* = 17.3195 (16) Å	*T* = 298 (2) K
*V* = 1610.2 (3) Å^3^	Prism, yellow
*Z* = 4	0.25 × 0.23 × 0.21 mm

### Data collection

**Table:**

Stoe IPDS II diffractometer	2157 independent reflections
Radiation source: fine-focus sealed tube	1779 reflections with *I* > 2σ(*I*)
Monochromator: graphite	*R*_int_ = 0.049
Detector resolution: 0.15 mm pixels mm^-1^	θ_max_ = 29.2º
*T* = 298(2) K	θ_min_ = 2.8º
rotation method scans	*h* = −6→9
Absorption correction: numericalshape of crystal determined optically	*k* = −18→17
*T*_min_ = 0.172, *T*_max_ = 0.275	*l* = −23→18
5803 measured reflections	

### Refinement

**Table:**

Refinement on *F*^2^	Secondary atom site location: difference Fourier map
Least-squares matrix: full	Hydrogen site location: inferred from neighbouring sites
*R*[*F*^2^ > 2σ(*F*^2^)] = 0.038	H-atom parameters constrained
*wR*(*F*^2^) = 0.097	*w* = 1/[σ^2^(*F*_o_^2^) + (0.0451*P*)^2^ + 3.1448*P*] where *P* = (*F*_o_^2^ + 2*F*_c_^2^)/3
*S* = 1.16	(Δ/σ)_max_ = 0.007
2157 reflections	Δρ_max_ = 0.96 e Å^−^^3^
87 parameters	Δρ_min_ = −0.81 e Å^−^^3^
Primary atom site location: structure-invariant direct methods	Extinction correction: none

### Special details

**Table:**

Experimental. (X-SHAPE and X-RED; Stoe & Cie, 2005)
Geometry. All e.s.d.'s (except the e.s.d. in the dihedral angle between two l.s. planes) are estimated using the full covariance matrix. The cell e.s.d.'s are taken into account individually in the estimation of e.s.d.'s in distances, angles and torsion angles; correlations between e.s.d.'s in cell parameters are only used when they are defined by crystal symmetry. An approximate (isotropic) treatment of cell e.s.d.'s is used for estimating e.s.d.'s involving l.s. planes.
Refinement. Refinement of F^2^ against ALL reflections. The weighted R-factor wR and goodness of fit S are based on F^2^, conventional R-factors R are based on F, with F set to zero for negative F^2^. The threshold expression of F^2^ > 2sigma(F^2^) is used only for calculating R-factors(gt) etc. and is not relevant to the choice of reflections for refinement. R-factors based on F^2^ are statistically about twice as large as those based on F, and R- factors based on ALL data will be even larger.

### Fractional atomic coordinates and isotropic or equivalent isotropic displacement parameters (Å^2^)

**Table:**

	*x*	*y*	*z*	*U*_iso_*/*U*_eq_	
Pt1	0.2500	0.2500	0.451996 (14)	0.02949 (11)	
Cl1	0.0431 (2)	0.38548 (12)	0.45575 (10)	0.0500 (4)	
Cl2	0.0689 (2)	0.17462 (12)	0.35700 (8)	0.0481 (3)	
N1	0.4010 (6)	0.3087 (3)	0.5416 (2)	0.0316 (9)	
C1	0.3349 (8)	0.2829 (4)	0.6127 (3)	0.0304 (10)	
C2	0.4230 (8)	0.3185 (4)	0.6782 (3)	0.0381 (11)	
H2	0.3744	0.3015	0.7265	0.046*	
C3	0.5850 (8)	0.3801 (4)	0.6728 (3)	0.0399 (12)	
C4	0.6800 (11)	0.4197 (6)	0.7439 (4)	0.0578 (18)	
H4A	0.7228	0.3649	0.7753	0.069*	
H4B	0.5898	0.4595	0.7725	0.069*	
H4C	0.7884	0.4601	0.7296	0.069*	
C5	0.6513 (9)	0.4028 (5)	0.5998 (4)	0.0472 (14)	
H5	0.7597	0.4429	0.5942	0.057*	
C6	0.5593 (9)	0.3668 (5)	0.5349 (3)	0.0424 (13)	
H6	0.6065	0.3827	0.4862	0.051*	

### Atomic displacement parameters (Å^2^)

**Table:**

	*U*^11^	*U*^22^	*U*^33^	*U*^12^	*U*^13^	*U*^23^
Pt1	0.03027 (15)	0.03300 (16)	0.02522 (16)	−0.00577 (10)	0.000	0.000
Cl1	0.0429 (7)	0.0431 (8)	0.0638 (10)	0.0050 (6)	0.0091 (7)	0.0049 (6)
Cl2	0.0523 (8)	0.0592 (8)	0.0327 (6)	−0.0138 (7)	−0.0124 (6)	−0.0019 (6)
N1	0.031 (2)	0.035 (2)	0.0283 (19)	−0.0121 (18)	−0.0011 (16)	−0.0041 (16)
C1	0.031 (2)	0.036 (2)	0.024 (2)	−0.011 (2)	−0.0050 (19)	0.0000 (18)
C2	0.040 (3)	0.044 (3)	0.031 (2)	−0.007 (2)	−0.002 (2)	0.004 (2)
C3	0.035 (3)	0.039 (3)	0.046 (3)	−0.011 (2)	−0.007 (2)	−0.001 (2)
C4	0.054 (4)	0.070 (4)	0.049 (4)	−0.021 (4)	−0.018 (3)	−0.005 (3)
C5	0.036 (3)	0.053 (4)	0.053 (3)	−0.020 (3)	−0.006 (3)	−0.003 (3)
C6	0.040 (3)	0.052 (3)	0.035 (2)	−0.020 (3)	0.007 (2)	−0.001 (2)

### Geometric parameters (Å, °)

**Table:**

Pt1—N1	2.031 (4)	C2—H2	0.9300
Pt1—N1^i^	2.031 (4)	C3—C5	1.380 (9)
Pt1—Cl2	2.3038 (13)	C3—C4	1.494 (8)
Pt1—Cl2^i^	2.3038 (13)	C4—H4A	0.9600
Pt1—Cl1^i^	2.3146 (16)	C4—H4B	0.9600
Pt1—Cl1	2.3146 (16)	C4—H4C	0.9600
C1—N1	1.359 (6)	C5—C6	1.379 (8)
C1—C2	1.374 (7)	C5—H5	0.9300
C1—C1^i^	1.472 (10)	C6—N1	1.352 (7)
C2—C3	1.398 (7)	C6—H6	0.9300
			
N1—Pt1—N1^i^	80.4 (2)	C3—C2—H2	119.7
N1—Pt1—Cl2	175.58 (12)	C5—C3—C2	117.4 (5)
N1^i^—Pt1—Cl2	95.40 (13)	C5—C3—C4	122.0 (5)
N1—Pt1—Cl2^i^	95.40 (13)	C2—C3—C4	120.6 (6)
N1^i^—Pt1—Cl2^i^	175.58 (12)	C3—C4—H4A	109.5
Cl2—Pt1—Cl2^i^	88.85 (8)	C3—C4—H4B	109.5
N1—Pt1—Cl1^i^	87.72 (14)	H4A—C4—H4B	109.5
N1^i^—Pt1—Cl1^i^	89.81 (14)	C3—C4—H4C	109.5
Cl2—Pt1—Cl1^i^	90.96 (6)	H4A—C4—H4C	109.5
Cl2^i^—Pt1—Cl1^i^	91.34 (6)	H4B—C4—H4C	109.5
N1—Pt1—Cl1	89.81 (14)	C6—C5—C3	121.0 (5)
N1^i^—Pt1—Cl1	87.72 (14)	C6—C5—H5	119.5
Cl2—Pt1—Cl1	91.34 (6)	C3—C5—H5	119.5
Cl2^i^—Pt1—Cl1	90.96 (6)	N1—C6—C5	120.6 (5)
Cl1^i^—Pt1—Cl1	176.78 (8)	N1—C6—H6	119.7
N1—C1—C2	120.6 (5)	C5—C6—H6	119.7
N1—C1—C1^i^	115.0 (3)	C6—N1—C1	119.9 (4)
C2—C1—C1^i^	124.4 (3)	C6—N1—Pt1	125.3 (3)
C1—C2—C3	120.5 (5)	C1—N1—Pt1	114.8 (3)
C1—C2—H2	119.7		
			
N1—C1—C2—C3	−1.5 (9)	C1^i^—C1—N1—C6	−178.3 (6)
C1^i^—C1—C2—C3	179.4 (7)	C2—C1—N1—Pt1	−179.3 (4)
C1—C2—C3—C5	−0.1 (9)	C1^i^—C1—N1—Pt1	−0.1 (8)
C1—C2—C3—C4	179.4 (6)	N1^i^—Pt1—N1—C6	178.1 (6)
C2—C3—C5—C6	0.7 (10)	Cl1^i^—Pt1—N1—C6	87.9 (5)
C4—C3—C5—C6	−178.8 (7)	Cl1—Pt1—N1—C6	−94.2 (5)
C3—C5—C6—N1	0.3 (10)	N1^i^—Pt1—N1—C1	0.0 (3)
C5—C6—N1—C1	−2.0 (9)	Cl2^i^—Pt1—N1—C1	178.7 (4)
C5—C6—N1—Pt1	−179.9 (5)	Cl1^i^—Pt1—N1—C1	−90.2 (4)
C2—C1—N1—C6	2.6 (9)	Cl1—Pt1—N1—C1	87.8 (4)

Symmetry codes: (i) −*x*+1/2, −*y*+1/2, *z*.
